# T Lymphocytes and Inflammatory Mediators in the Interplay between Brain and Blood in Alzheimer's Disease: Potential Pools of New Biomarkers

**DOI:** 10.1155/2017/4626540

**Published:** 2017-02-15

**Authors:** Anna Mietelska-Porowska, Urszula Wojda

**Affiliations:** Laboratory of Preclinical Testing of Higher Standard, Nencki Institute of Experimental Biology, Pasteur 3, 02-093 Warsaw, Poland

## Abstract

Alzheimer's disease (AD) is a chronic neurodegenerative disorder and the main cause of dementia. The disease is among the leading medical concerns of the modern world, because only symptomatic therapies are available, and no reliable, easily accessible biomarkers exist for AD detection and monitoring. Therefore extensive research is conducted to elucidate the mechanisms of AD pathogenesis, which seems to be heterogeneous and multifactorial. Recently much attention has been given to the neuroinflammation and activation of glial cells in the AD brain. Reports also highlighted the proinflammatory role of T lymphocytes infiltrating the AD brain. However, in AD molecular and cellular alterations involving T cells and immune mediators occur not only in the brain, but also in the blood and the cerebrospinal fluid (CSF). Here we review alterations concerning T lymphocytes and related immune mediators in the AD brain, CSF, and blood and the mechanisms by which peripheral T cells cross the blood brain barrier and the blood-CSF barrier. This knowledge is relevant for better AD therapies and for identification of novel biomarkers for improved AD diagnostics in the blood and the CSF. The data will be reviewed with the special emphasis on possibilities for development of AD biomarkers.

## 1. Introduction

Alzheimer's disease (AD) is a chronic neurodegenerative disorder and the most common cause of dementia, manifesting as the progressive loss of memory and cognition. Modern statistics predict that, due to the lengthening lifespan, about 115 million people will be affected by this disease to the year 2050 [1]. The World Alzheimer Report indicates that AD and other dementia diseases are the cause of a progressive epidemic, which may be “the world's greatest health and social crisis” [2].

In AD the gradual death of nerve cells occurs in brain regions responsible for learning and cognition (cerebral cortex), memory and spatial orientation (hippocampus), and emotion (amygdala). However, AD develops for tens of years without clinical dementia symptoms. Clinical AD begins with the early and mild dementia stage in patients with mild cognitive impairment (MCI) and within an average of 10 years gradually progresses to moderate and later to severe AD. Due to the lack of sufficient knowledge on the mechanisms which initiate and drive the development of AD, patients affected by this disease are treated only with symptomatic therapies. The lack of early AD biomarkers, preferably from the more accessible tissues than brain, greatly hinders the treatment of AD, which is usually introduced only in the relatively late, already developed clinical stage of the disease. Moreover, biomarkers which would help in monitoring the AD progression and response to therapies are also missing. Therefore there is an intensive, ongoing search for drugs, which would treat not only the symptoms, but primarily the cause(s) of AD, and for biomarkers, preferably from the accessible tissues such as blood or the CSF. Winning this war requires elucidating the mechanisms of AD pathogenesis.

The mechanisms of AD pathogenesis and progression so far remain unclear. The search for AD etiology began with an analysis of postmortem degenerative changes in patients' brains. As described for the first time by Alois Alzheimer in 1906, the AD brain is characterized by the presence of two main pathological hallmarks: amyloid plaques, which are extracellular aggregates of an amyloid-beta (A*β*) peptide [3], and intraneuronal neurofibrillary tangles (NFT), which are mainly composed of hyperphosphorylated tau protein [4]. Moreover, Alzheimer already described the inflammatory features of the AD brain. Both A*β* plaques and NFT are to this day a very important criterion for the histological diagnosis of AD postmortem, which includes the assessment of so called neuritic (senile) plaques, extracellular protein aggregates comprised of a core of amyloid fibrils, dystrophic neurites, and glial cells (astrocytes and microglia). The main component of the deposits in senile plaques is the A*β*42 amyloid protein [5].

Based on neuropathological AD hallmarks, the most well-known hypothesis concerning AD pathogenesis is the amyloid cascade hypothesis, which assumes that a key event in the development of this pathology is an abnormal amyloidogenic cleavage of the transmembrane amyloid precursor protein (APP). This leads to the overproduction, oligomerization, and later on the deposition of A*β* protein aggregates in the central nervous system (CNS); in turn, the oligomerization of A*β* is thought to initiate a sequence of events resulting in the degeneration of neuronal synapses and neurotransmission disorders (mainly cholinergic) and which results in inflammation and the death of large populations of neurons. In particular, the appearance of pathological forms of A*β* is considered to activate inflammatory processes involving microglia and astrocytes, oxidative stress, tauopathy, and synaptic loss, which ultimately leads to cognitive impairment [6]. The amyloid cascade hypothesis was strongly supported by the identification of rare early-onset familial AD cases (<5% of patients) linked to mutations in the genes encoding APP or enzymes involved in APP amyloidogenic cleavage, presenilins 1 and 2 (PS1 and PS2). However, the etiology of the most common, late-onset AD form (spontaneous) is much less known [7, 8]. The amyloid cascade hypothesis has served as a foundation for the development of new therapeutic strategies for AD [9–15], but drugs targeting A*β* have so far failed in clinical trials. This raises the question whether approaching AD therapy as if it were a monofactorial disease, caused and driven by A*β*, was right.

Mounting evidence indicates that mechanisms responsible for AD pathogenesis and progression are heterogeneous and multifactorial, especially in the case of the most common sporadic form of AD (SAD) (reviewed in [16, 17]). While the important role of A*β* in AD pathology is well-proved, according to the current view, progress in the causal treatment and in diagnostics of AD critically depends on the clarification of complex molecular mechanisms of AD pathogenesis. This approach can enable identifying new drug targets and novel biomarkers for improved AD diagnostics. Among processes and factors which have been found as contributing to AD pathogenesis and progression are inflammatory processes [18–21], abnormal glucose metabolism [22–25], increased oxidative stress and mitochondria impairment [21, 26–29], dysregulation of apoptosis and cell cycle [29–31], disorders of calcium homeostasis [32–34], abnormal cholesterol homeostasis [35–37], and dysfunction of synapses [38, 39].

A lot of attention has been given to the inflammation hypothesis in AD, and several excellent general reviews on the role of inflammation in the AD have been published in recent years [40, 41]. The main focus has been given to activation of glial cells in AD brain and a possibility of immunotherapy in AD has been broadly discussed. Recently it was demonstrated that the induction of the interferon- (IFN-) *γ*-dependent recruitment of monocyte-derived macrophages to the brain resulted in the clearance of cerebral amyloid-*β* (A*β*) plaques and improved cognitive performance in a mice AD model [42]. These findings significantly support the rationale for immunotherapy in AD [42, 43].

In addition to glial cells, several recent reports also highlighted the role of peripheral T lymphocytes in the innate immunity of AD neuroinflammatory processes, as reviewed in [44]. In order to get to the brain, T lymphocytes have to pass through two main barriers: the blood-cerebrospinal fluid barrier (BCSFB) and the blood brain barrier (BBB). In this process, as well as in the inflammatory response in AD, immune mediators play a key role. A systematic review of the mechanisms involving T cells and immune mediators in AD and the interplay between their pools in the brain, the CSF, and the blood has been missing. In this article we set to fulfill this gap. We summarize the findings on the migration of peripheral blood T lymphocytes through the BCSFB and the BBB to the AD brain and on the interactions of the immune mediators and T lymphocytes between the brain, the CSF, and the blood in AD. This knowledge is relevant not only for elucidating the lymphocytes' role in AD pathomechanisms, but also for studies exploring easily accessible blood and the cerebrospinal fluid (CSF) in search for novel biomarkers for the early diagnosis of AD.

## 2. Neuroinflammation in the AD Brain

According to the current view, the innate immune system plays a dominating role in AD inflammation (reviewed in [40, 41]). The innate response is mediated by immune cells that are recruited to the site of an injury as a result of the initial signaling of inflammatory mediators cytokines and chemokines produced in the AD brain by destroyed neurons and astroglial and microglial cells [45, 46]. During the development of AD pathology, degenerating cells, as well as abnormal inclusions of A*β* and tau proteins, can act as proinflammatory factors [47]. The deposition of A*β* in the brain is associated with the activation of glial cells, astrocytes, and microglia. Microglial cells internalize soluble A*β* isoforms through phagocytosis, while insoluble A*β* deposits can activate microglia by binding to Toll-like receptors (TLRs). In microglia cells, A*β* activates mitogen-activated protein kinases (MAPKs) and stimulates proinflammatory gene expression that leads to the secretion of cytokines and chemokines, contributing to the inflammation process in the AD brain [48]. Chemokines secreted by brain cells can attract blood-derived immune cells, which can infiltrate the brain due to impairments in the two main barriers: the BCSFB and the BBB. Thus, not only brain cells and cell-derived mediators are involved in inflammation during AD pathogenesis. Existing data indicates that lymphocytes are recruited from peripheral blood into CNS and contribute to an immune reaction in response to pathological conditions [49].

Many reports confirmed the special role of T cells in the development of neuroinflammation in AD [50–56]. The specific role of T cells in AD brain pathology was indicated by demonstrating increased levels of peripheral T cells in postmortem brains from AD patients in comparison to brain tissues from other neurodegenerative disorders [57]. T cells cooperate with and modulate the innate immune system and seem to be important in protection against AD. However, in AD, the role of A*β*-specific T cells is difficult to accurately determine because of the bidirectionality of their functions; it is known that they may act in either protective or damaging roles. It is believed that T cells specific for A*β*1-40 peptide can prevent the development of A*β* plaques because their presence has been detected mainly in healthy individuals and not in AD patients. In contrast, T cells specific for A*β*1-42 are detectable in AD individuals, which indicates that they may play a role during the plaques formation [58]. Moreover, A*β*-specific Th1 cells induce the production of proinflammatory cytokines by microglial cells, and A*β*-specific Th2 cells possess the properties of inhibiting the production of cytokines by glial cells [59]. It is possible that different stages of AD progression have distinct profiles of T cell subpopulations and that the immune cells may play contradictory roles at the early AD versus later AD stages. More studies are needed to verify this possibility.

Further we will focus on the mechanisms by which peripheral T lymphocytes are mobilized from the blood to the brain and how they cross the BCSFB and the BBB and on the participation of immune mediators in the blood, the brain, and the CSF in the inflammatory process involving T cells in AD.

### 2.1. T Lymphocytes and Immune Mediators at the AD Blood Brain Barrier

**Figure 1:**
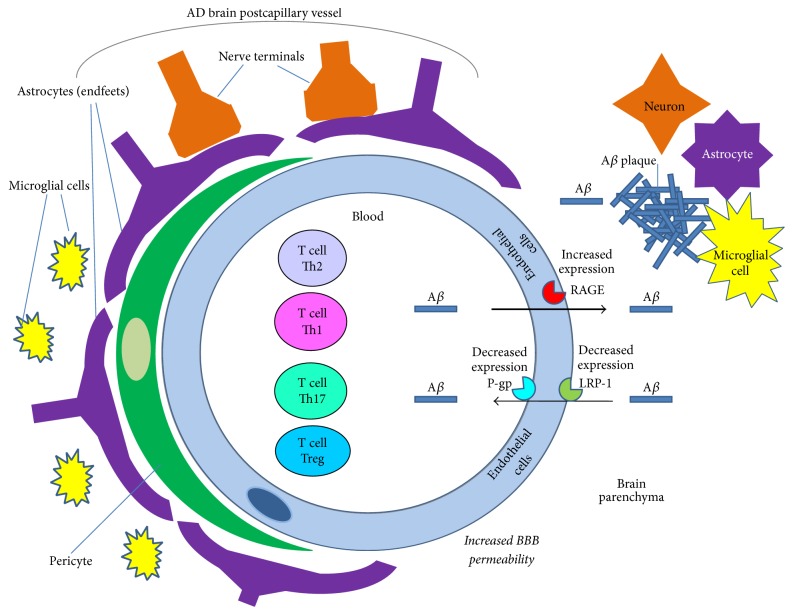
A*β* transport across the blood brain barrier (BBB) in Alzheimer disease. In Alzheimer disease the loosing of tight junctions leads to increased BBB permeability. In healthy conditions the A*β* peptide is transported to the brain by the receptor for advanced glycosylation products (RAGE) and cleared from the brain to the blood by LDL-Receptor-Proteins (LRPs) followed by p-glycoprotein (P-gp). In Alzheimer's disease these transport systems are impaired: the expression of RAGE is increased and the expression of LRPs is decreased, leading to the accumulation of A*β* in the brain.

**Figure 2:**
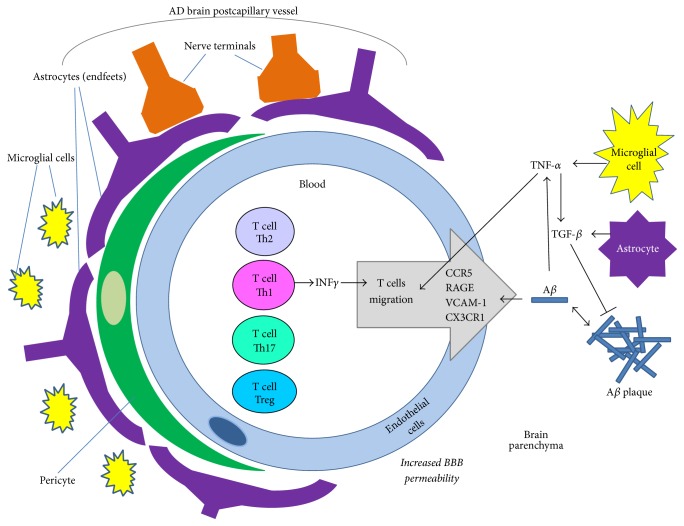
T lymphocytes migration at the blood brain barrier (BBB) in AD. The key role of the BBB is defending the brain against the harmful influence of peripheral factors. In AD the permeability of the BBB is increased, and the migration of peripheral immune cells contributes to the progress of neuroinflammation in the brain. In response to A*β* stimulation from the parenchyma, the transmigration of immune cells from the blood into the brain is mediated by VCAM-1. At postcapillary vessels the immune cells cross the BBB upon interaction with the CX3CR1 receptor on endothelial cells. Other key mediators of the immune cells' migration are the RAGE receptor and the CCR5 chemokine receptor. Moreover, A*β* activates microglial cells to produce TNF-*α*, which can promote transendothelial migration of T cells and has the ability to enable astroglial cells' activation and overproduction of TGF-*β*1, which in turn may lead to decreased A*β* plaque loads.

In physiological conditions the brain employs specific immune control mechanisms to precisely regulate inflammation processes and counteract brain damage [49]. The key role in the brain's defense against the harmful influence of peripheral factors is played by the BBB [60]. The BBB is created from neurovascular units composed of endothelial cells bound by tight junctions, wrapped by basement membranes, and surrounded by pericytes and astrocytic endfeet [61]. Its general function is not only restricting pathogens and macromolecules from entering the brain, but also circulating cells. The BBB's increased permeability and the infiltration of circulating immune cells from the periphery into the CNS are characteristic for several neurodegenerative conditions, including AD [62, 63]. AD-specific features of the BBB are schematically summarized in Figures 1 and 2. One of AD's specific features is cerebral amyloid angiopathy (CAA), an increased accumulation and deposition of A*β* in neurovascular units, which starts from A*β* accumulation at the outer basal lamina and results in the impairment of the BBB [63]. There are two main mechanisms of CAA development, as shown schematically in Figure 1. The first one is an increased efflux of A*β* into the brain parenchyma due to the increased expression of a receptor for advanced glycation end products (RAGE) by endothelial cells. RAGE is implicated in AD pathology by mediating transport of pathologically relevant concentrations of A*β* from the blood into the CNS by inflammatory and procoagulant responses in the endothelium. After crossing the BBB, circulating A*β* is rapidly taken up by neurons inducing cellular stress, whereas RAGE/A*β* interaction in the brain endothelium results in the suppression of blood flow [64]. The second mechanism is associated with the failure of A*β* clearance. This process is mediated by a decreased level of the low-density lipoprotein receptor-related protein-1 (LPR-1) expressed at the abluminal surface of the brain's endothelial cells and a decreased level of p-glycoprotein (P-gp) in the luminal plasma membrane of the brain capillary endothelium, what leads to a decreased efflux of A*β* from the brain into the blood [65, 66] (Figure 1).

The migration of peripheral immune cells into the brain occurs at postcapillary vessels and is a multistage mechanism. After the initial binding to selectins on endothelial cells, the immune cells roll along the endothelium and, upon interaction with the chemokine receptor CX3CR1 expressed on endothelial cells, the immune cells circulate against the direction of blood flow [67, 68]. Interaction with endothelial CX3CR1 leads to the activation of integrins on the surface of immune cells and to the adhesion of immune cells to the endothelium, followed by transendothelial migration of immune cells or extravasation from the blood vessel (Figure 2). After crossing the endothelium, the immune cells migrate directly towards the inflammation site, crossing the perivascular space [67].

During AD development, brain amyloidosis stimulates the expression of vascular adhesion molecules in brain vessels that enhance the transmigration of immune cells [69]. All types of peripheral immune cells may infiltrate through the pathologically altered BBB during AD pathogenesis. Among them are T cells [70]. There is evidence that dendritic cells (CD11+) can stimulate the infiltration of A*β*-specific T cells to target A*β* deposition in the brain [71]. Other studies showed that A*β*1-42 activates the microglial release of TNF-*α*, which can promote the transendothelial migration of T cells [72], and can also increase the local expression of TGF-*β*1 in astrocytes. Overproduction of TGF-1*β* can in turn decrease A*β* accumulation and plaque formation (Figure 2) but also has the ability to increase cerebrovascular amyloidosis and thus lead to enhanced neuroinflammation [73]. It seems that T cells contribute to the removal of toxic A*β* species and enhance inflammation. A decreased crosstalk between T cells and endothelial cells leads to the decreased activation of T cells and the inhibition of the A*β* phagocytosis process in the vicinity of the BBB [74]. McManus and coworkers showed that infiltrated T cells produce IFN-*γ* and IL-17, which are involved in glial activation, exacerbating neuroinflammation [75]. The etiology of AD is very complex and possibly involves infectious agents, among other factors. McManus and collaborators in their studies on the influence of infection on AD pathology confirmed that not only Th1 cells but also Th17 cells infiltrate from the blood into the brain during AD progression [75]. They described that in APP/PS1 mice peripheral infection with a common human pathogen* (Bordetella pertussis)* induces IFN-*γ*+ Th1 cells and IL-17+ T17 cells infiltration into the brain. They also confirmed that this process is age-dependent and older APP/PS1 mice are more susceptible to this effect than younger. A significant number of Th1 and Th17 cells were identified in the brains of 12-month-old APP/PS1 mice and this was additionally accompanied by increases in glial activation and A*β* accumulation. The particular role of IL-17+ T cells in AD pathogenesis is still debated, though it has been reported that there is a skewing of T cells in AD to a Th17 phenotype. It is suggested that the A*β*-specific Th1 cells and Th17 cells together may lead to microglial activation and inflammatory changes in the brain (Figures 1 and 2).

As described above, the interplay between all components of the immune response including T lymphocytes in AD is dynamic and complex but significantly contributes to the exacerbation and progression of AD neuroinflammation.

Cytokines, chemokines, and their receptors play a prominent role in the infiltration of immune cells through the BBB. Endothelial cells per se are involved in the transport of cytokines (TNF*α*) and chemokines (CCL11) from the brain to the blood and reversely [76]. Furthermore, chemokines and their receptors expressed by T cells are involved in the migration of T cells crossing the endothelium in AD patients. The main function in this process is assigned to chemokine receptors MIP-1*α* and CXCR2. Additionally MIP-1*α* may also bind to CCR5 expressed in brain endothelial cells during the late stage of AD, leading to T cell migration through endothelial tight junctions into the CNS. Studies in AD model animals indicated that A*β* upregulates RAGE and CCR5, which in turn leads to the promotion of T cell migration into the brain (Figure 2) [77].

### 2.2. T Lymphocytes and Immune Mediators at the AD Blood-Cerebrospinal Fluid Barrier

**Figure 3:**
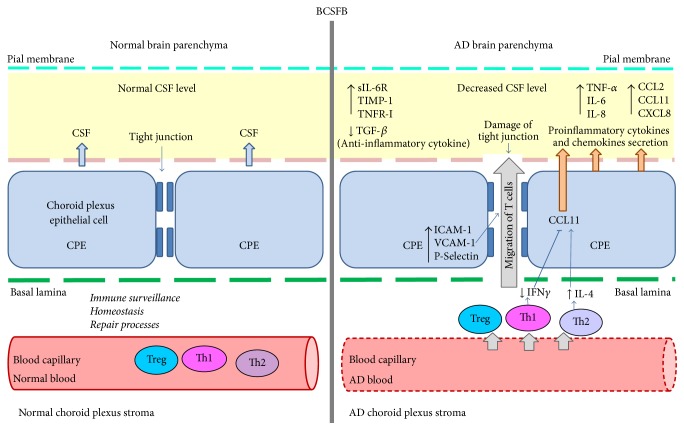
T lymphocytes infiltration and inflammatory mediators at the blood-cerebrospinal fluid barrier (BCSFB) in AD. The BCSFB is a selective barrier that restricts the passages of circulating immune cells from the stroma compartment into the brain parenchyma. In AD the BCSFB is impaired and T cells migrating through the BCSFB produce inflammatory mediators which are associated with the immune response to pathological conditions. At the BCSFB the chemokine CCL11 is produced in response to IL-4 secreted from Th2 cells and can be inhibited by IFN-*γ* secreted from Th1 cells. In the CSF in AD the increased levels of cytokines: TNF-*α*, IL-6, IL-8, chemokines: CXCL8, CCL11, and CCL2, and cytokine receptors: sIL-6R, TIMP-1, and TNFR-I are observed. Moreover, enhanced expression of ICAM-1, VCAM-1, and P-selectin in the CPE leads to the damage of tight junctions and modulates immune cell migration to the CSF. Additional explanations can be found in the article text. Bold arrows indicate regulation of molecule levels [↑, upregulation, ↓, downregulation]. Blue arrows indicate affected processes.

The blood and the CSF are intensively explored in search for biomarkers which could enable the early diagnosis of AD. Therefore currently a lot of research focuses on the structural and functional alterations in the blood-cerebrospinal fluid barrier (BCSFB) and changes in CSF composition during pathological processes in the CNS. The BCSFB is also actively involved in neuroinflammatory processes. The BCSFB is formed from the choroid plexus (CP) and is composed of a single continuous layer of modified cuboidal epithelial cells (CPE), which are attached to the basal lamina. CPE are bound to each other by tight junctions formed by claudins and occludins [78] (Figure 3). The remaining part of the BCSFB is the arachnoid membrane, which envelops the brain. The cells of this membrane are also linked by tight junctions. The major site of CSF formation is the layer of CPEs of the CP [79]. Under physiological conditions, the BCSFB is a selective barrier that restricts the passage of molecules and cells, including circulating immune cells, from the stromal compartment into the brain parenchyma [80, 81], and plays an essential role in immune surveillance and the maintenance of homeostasis and repair processes in the brain. The BCSFB fulfills these functions by secreting and regulating the levels of trophic factors and cytokines [80, 82, 83]. The BCSFB acts as a relay station that senses inflammation signals from both the CNS [84, 85] and the periphery [86, 87]. CPE of the BCSFB contain many transport systems and receptors that enable them to regulate transport from the blood to the CSF and reversely. These cells have an ability to respond to inflammatory stimuli by producing proinflammatory molecules. This may result in the impairment of the barrier's integrity and the transmission of inflammatory signals to the brain [88, 89]. Later this leads to the increased influx of white blood cells from the bloodstream into the brain parenchyma [90]. The process of the recruitment and entry of immune cells through the CP is mediated by the expression of intercellular adhesion molecule 1 (ICAM-1), vascular cell adhesion molecule 1, and P-selectin [91]. The AD features of the BCSFB are schematically summarized in Figure 3. During AD pathogenesis, structural and functional alterations in the CP are observed, which results in a decreased CSF production and changes in metabolic activity [92, 93]. Current data indicates that the CP plays a central role in the neuroinflammatory response, both in AD and in aging [94, 95]. Age-dependent alterations in the secretion of inflammatory molecules by CP cells are viewed as a key factor associated with deficits in brain cell functions and plasticity and with alterations in glial activation, neurogenesis, and cell survival [96–98]. Marques and collaborators report that the inflammatory response of CP epithelial cells is associated with the overexpression of gene encoding lipocalin-2 (LCN2), which is involved in the rapid proinflammatory response of the innate immune system [99]. Furthermore, it has been suggested that LCN2 participates in regulating the neuroinflammatory response to an increased level of A*β* peptides and an oxidative stress insult and plays a role in the modulation of brain cell activation, migration, and survival [100, 101]. Monomeric and oligomeric A*β* forms can activate microglial cells to secrete proinflammatory cytokines in the brain [102]. Brkic and collaborators reported that soluble A*β*1–42 oligomers injected into the cerebral ventricles of mice increased the levels of cytokines and chemokines in the CSF without microglia activation, which indicates that in the early stage of AD the inflammatory signal generated in CPE's in response to soluble A*β*1–42 oligomers and impairments in the BCSFB might occur before microglia activation [89]. This supports the notion that the immune system plays an important role not only at the late AD stages, but also at the early step of the pathology development, and thus that early AD markers may be identified among the immune mediators.

Recently, Villeda and coworkers using a mouse animal model confirmed that an increased level of the CCL11 chemokine is associated with age-dependent peripheral systemic alterations that affect neurogenesis processes in the hippocampus and memory formation [103]. Erickson and collaborators demonstrated that CCL11 can cross into the brain through direct interaction with the BBB [76]. Additionally, some data suggests that an increased level of CCL11 is involved in changes in the BCSFB. Firstly, this is due to the overexpression of gene encoding CCL11 in CP cells, and secondly CCL11 is produced in CP cells in response to IL-4 secretion. Both ways lead to a significant rise of the level of CCL11 in the CSF. Baruch and coworkers also showed that INF-*γ* can inhibit IL-4-dependent CCL11 production in CP cells. Moreover, they reported the increase of IL-4 and decrease of INF-*γ* levels in CP during the aging process, associated with the alteration of T cells recruitment from the blood into the stroma [95, 96] (Figure 3).

In order to investigate the mechanisms involved in the neuroinflammatory signature of AD, Delaby and collaborators performed a multiplex analysis of human samples of CSF and blood serum from 55 individuals and evaluated the expression level of 120 potential AD biomarkers corresponding to cytokines and chemokines and other signaling proteins. Based on their observations more characteristic changes were found related to cytokines receptors than cytokines per se. For example, they observed a significant increase in the level of sIL-6R, TIMP-1, and TNFR-I receptors in the CSF. Furthermore, although it is evaluated that the exposure of microglia to A*β* deposits increases the levels of IL-6 in the CSF and serum [104], Delaby et al. observed a significantly raised level only of sIL-6R. Similarly, an increased level of TNF-*α* and a decreased level of TGF-*β* in the CSF were previously described as markers of the conversion of MCI to AD [105]. However Delaby et al. reported no changes in the level of these cytokines, but they did report a significantly increased level of sTNFR-I in the CSF and a decreasing tendency in the serum of AD patients compared to controls (Figure 3). Their analysis indicates that sTNF-RI and TIMP-1 and sILR-6 are promising AD biomarker candidates. Moreover, they noted an increased level of IL-8 and CCL2 (MCP-1) in the CSF. However, contrary to the results from blood analysis, they described no changes in CCL5 and CXCL3 levels in CSF samples from AD patients in comparison to controls. Because some of these results are contrary to data from previous studies, Delaby suggests that in following tests CSF putative biomarkers should subsequently be tested for confirmation in an enlarged cohort and through more sensitive quantitative analysis [106]. Additionally, Kauwe and coworkers, based on data from genome-wide association studies (GWAS) and in accordance with other previous findings, also confirmed the increased level of CCL2 in CSF samples from patients with prodromal AD, which correlated with a faster cognitive decline [107, 108].

Summarizing, neuroinflammation is one of the hallmarks of AD and according to current knowledge inflammatory mediators present in the CSF and blood, particularly cytokines and chemokines, may represent biomarkers for disease screening in patients with severe AD, as well as those with mild cognitive impairment, and may help to diagnose the early stages of AD.

### 2.3. T Lymphocytes and Inflammatory Mediators in the Interplay between AD Blood and Brain

In AD brain, among the most important cytokines of the immune response, IL-1, IL-4, IL-6, IL-10, IFN-*γ*, and TNF-*α* are reported most often [109, 110]. During pathological process microglial cells produce some of these proinflammatory and anti-inflammatory cytokines via direct interaction with infiltrated T lymphocytes [111]. The pathogenic reaction of T cells against A*β* is initiated by the entry of a cluster of differentiated (CD) 8 cytotoxic T cells into the brain, followed by the secretion of proinflammatory cytokines by CD4+ cells [112, 113].

Referring to T lymphocytes and related immune mediators in AD, such data is predominantly derived from in vitro studies, which use cell lines isolated from the blood of AD patients and from in vivo studies involving animal AD models. In in vitro conditions the stimulation of peripheral blood mononuclear cells (PBMCs) by A*β*42 induces these cells to produce cytokines and chemokines. This study showed a significantly high production of the inflammatory cytokines IL-1*β*, IL-6, TNF-*α*, and IFN-*γ* in response to A*β*. This observation suggests that PBMC cells are important players in the inflammatory response of AD pathogenesis [114]. Similarly, Martorana and coworkers reported an increase of the anti-inflammatory cytokine IL-10 and IL-1 receptor antagonist in the PBMCs in in vitro study and they hypothesized that this situation might balance the overproduction of the above-described proinflammatory cytokines. The additional effect of the amyloid efflux from the brain to the blood, which can prime lymphocytes in physiological conditions, should be taken into consideration [115]. On the other hand, an overexpression of IL-1 in hippocampal neurons from AD patients is shown at all stages of tau protein neurofibrillary tangle formation [116, 117], as well as microglia-derived IL-1 association with A*β* plaques formation in brain parenchyma [118]. Consistently, microglial activation and IL-1 overexpression have been shown in transgenic animal models of AD [119]. Mrak and Griffin [120] also reported increased level of microglia-derived IL-1 in AD, which drive a cascade of development of amyloid plaques and neurofibrillary tangles. The role of IL-1 gene variants in these processes and its influence on changes of blood level of IL-1 as a potential biomarker of AD was indicated. However, the confirmation of this possibility seems to be difficult because of additional complications due to the influence of hypothalamic-pituitary-adrenal axis activity on peripheral cytokine production. In contrast, it was reported that decreased level of IL-1 in AD patients corresponds with an increase in AD severity [120]. Other evidence indicates that IL-1 leads to the increased expression and activity of neuronal acetyl cholinesterase, which could explain the cholinergic dysfunction characteristic of AD patients [121]. This issue requires further study.

Another important proinflammatory cytokine is IL-6, the secretion of which could be induced by IL-1*β* [122]. Based on the analysis of AD patients, the level of IL-6 in blood was significantly higher in the AD group versus the control group, and that increase was even more significant among older AD individuals [123]. This indicates that according to the characteristics of AD, changes in the level and expression of immune mediators in the blood may also be age-related. Butovsky and collaborators presented data that cytokines characteristic for T cells like IFN-*γ* and IL-4 can induce microglial cells to neuroprotective activity in response to an aggregated beta-amyloid. Interleukin-4 plays a protective role through the downregulation of TNF-*α* and upregulation of insulin-like growth factor I (IGF I). These findings suggest that the beneficial or harmful expression of the local immune response in the damaged CNS depends on the interplay of T cells and microglia [124]. In the Chao and coworkers in vitro study, pretreatment of cell cultures with IL-4 prevented neuronal cell injury induced by activated microglia [125]. According to Lee and coworkers IL-4 concentration is lower in the blood from AD patients, as compared to a control group [126]. Jabbari Azad and coworkers confirmed that a decrease of the IL-4 concentration level in blood, but also a significant increase of the IFN-*γ* level, correlated with the MMSE results of AD patients [123]. In Belkhelfa and collaborators in vivo study, IFN-*γ* and TNF-*α* levels in peripheral blood assessed in patients with AD in mild and severe stages, respectively, were higher than those observed in patients with moderate stage AD and MCI. An increased level of INF-*γ* observed in mild cases of AD indicates that it could serve as one of the markers of the early stage of the disease [127]. Moreover, INF-*γ* plays a key role during promoting processes of the immune reaction in the brain and has the ability to facilitate T cell migration [128]. On the other hand, INF-*γ* is also known as a bidirectional factor, which may prevent amyloid deposition during inflammatory process [129] but also enhance A*β* deposition through *β*-secretase 1 expression. Moreover, it was evaluated that TNF-*α* and IL-1*β* have the ability to increase the activity and/or expression of *γ*- and *β*-secretases [130, 131], which leads to A*β* deposition. Additionally IL-1*β*-expressing microglia are associated with A*β* plaques and NFT in the brain, where they correlate with progressive neuronal damage [132]. McQuillan and coworkers evaluated that A*β*-specific Th1 cells enhanced the A*β*-induced activation of microglia; they found that Th1 cells enhanced soluble and insoluble A*β* concentrations in the brains of APP/PSI mice [59].

Regulatory T cells (Treg) are also involved in the cytokine cascade during AD pathogenesis. Studies in Treg-depleted APP/PS1 mice, which are characterized by reduced microglia recruitment, suggest that Treg may contribute to the promotion of type 1 IFNs, depending on microglia activation in response to amyloid deposition [133]. Additionally the data from Dansokho et al. suggests that type 1 IFNs may help to restrain the development of the microglia proinflammatory activation profile at early stages of AD [133]. Other studies indicate that Treg can mediate their suppressive function through several effector mechanisms, including the production of immunosuppressive cytokines such as IL-10 and TGF-*β* [134, 135]. Cytokines such as IL-1, IL-4, IL-6, IL-10, IFN-*γ*, TNF*α*, and TGF-*β* can be taken into account in the search for new biomarkers of the early stages of AD [136].

Chemokines are chemotactic cytokines which stimulate and control the movement of leucocyte migration from the blood into the tissues. They play an important role in inflammation formation and are involved in the pathogenesis of many diseases [137–141]. The action of chemokines is determined by the expression of different surface receptors of cells from myeloid and lymphoid lines that lead to a chemotaxis [138]. The expression of chemokines and their receptors may be positively or negatively regulated at the transcriptional level by different factors, that is, proinflammatory cytokines, hypoxia, pathogens, stress, foreign antigens, and T cell costimulation [142–144]. The role of chemokines is to attract leukocytes to sites posing a threat, for example, infection. To reach the inflammation site circulating leukocytes must leave the bloodstream and enter the endothelium [145]. Proinflammatory cytokines such as IL-1 and TNF can affect the secretion of the proinflammatory chemokine belonging to the innate response, for example, CXCL8. Interferons and anti-inflammatory cytokines, that is, IL-10, can inhibit gene expression of inflammatory chemokines [146]. After antigen-specific cell activation, proinflammatory chemokines attract antigen-specific effector T cells to the inflammatory focal point. At the same time, Treg are recruited, and the balance between regulatory and effector cells determines the outcome of the local inflammatory process [147]. Furthermore, data indicates that chemokines play a role in starting the inflammatory response cascade and the recruitment of Treg [148].

The most commonly evaluated chemokine involved in AD neurodegenerative processes is CCL5 (RANTES), which regulates the expression and secretion of normal T cells. During AD pathogenesis an elevated level of the astroglial CCL5 chemokine is observed in the microcirculatory system of the brain [149]. CCL5 is upregulated as a response to a cytokine-mediated increase of reactive oxygen species (ROS) and oxidative stress in endothelial cells in the brain [150]. Its elevated levels contribute to the recruitment of immune-competent cells, which occurs concurrently with increased rates of neuronal deaths [151]. The CXC8 (IL-8) chemokine is produced in response to the proinflammatory signaling of A*β* by microglial cells. It plays a key role in phases with prevalent neurodegeneration through the recruitment of activated microglia into damaged areas of the brain during late stages of AD [47]. Other chemokines involved in AD pathogenesis are, for example, CCL2 (MCP-1); when its level increases, it results in the recruitment of activated monocyte cells [152, 153]. CX3CL1, also known as fractalkine, is produced in neurons and has the ability to control neurotoxicity through suppressing microglial activation by CX3CR1 receptor binding. An increased level of plasma-soluble CX3CL1 is observed in mild to moderate AD [154], which may indicate its potentially neuroprotective function. CCR2 and CCR5 chemokine receptors are expressed on lymphocytes. Martorana and coworkers reported an increased expression of both CCR2 and CCR5 receptors only in T cells after in vitro stimulation by A*β*42, whereas after the same treatment B cells only overexpress CCR5 [115]. It has been demonstrated that the CCL2 chemokine via CCR2 receptor, expressed on brain endothelial cells, contributes to increased brain endothelial permeability [155, 156]. Li and coworkers reported that A*β* interaction with RAGE receptor upregulates brain endothelial CCR5 expression and promotes T cells crossing the blood brain barrier [77, 157]. Moreover, peripheral T lymphocytes of AD patients are characterized by an increased level of CCL3 (MIP-1*α*) in comparison to healthy controls. In AD patients T cells infiltration may also result from an increased expression of the CXCR2 receptor [158]. While further research is needed to elucidate precise mechanism of AD at different stages of inflammatory response, these results all point to the important function of chemokines and their receptors in AD pathology and confirm their potential as biomarkers in the blood.

## 3. Dendritic Cells Contribution to T Cell Functions and Infiltration into the Brain

Nowadays it is increasingly accepted that inflammation is not only a consequence of neurodegeneration but also one of the major causative factors of AD, especially in its sporadic form [159]. Brain immune cells have an impaired ability to clear pathological forms of dangerous proteins that lead to the inflammation process and neuronal damage. In turn the ongoing inflammation in the brain enhances the progression of degeneration [41]. This phenomenon involves bidirectional communication between the CNS and systemic immunity where the recruitment of bone marrow-derived immunocompetent cells from systemic circulation to the brain is an important event during the progression of neurodegeneration [160, 161]. In addition to the T cells infiltration from the periphery into the AD brain and the T cell role in both brain venules and brain parenchyma, other immune cells which crosstalk with T cells, such as dendritic cells (DCs), as well as other myeloid cell types [68], also play an important contribution to neuroinflammation. Recently described CD33 and TREM2 genes, strictly coupled with myeloid cells, have been reported as significant risk factors associated with both familial and sporadic forms of AD. It is confirmed that these genes expressed both on microglial cells and peripheral myeloid cells may contribute to the clearance of misfolded pathological molecules [162, 163].

DCs maintain immune surveillance in neuroinflammation and neurodegeneration in the brain. Colton's studies [164] confirmed the presence of DCs in the CNS. These cells are present in the CSF, meninges, choroid plexus, and perivascular spaces [165] but they derive from circulating DC precursors, not from the brain. DCs have migratory abilities and play a crucial regulatory role in both innate and acquired immunity. These cells express major histocompatibility complex class II molecules (MHCII) and leukocyte integrin CD11c. DCs also play an important role in the communication between the brain and the periphery as a response to pathological conditions, when their amount in the CNS is significantly increased. DCs crosstalk with T cells and stimulate T cells during their migration from the blood to the brain in AD pathogenic processes [165, 166]. According to studies by Fisher and coworkers, DCs may regulate A*β*-specific T cell entry into the brain at perivascular and leptomeningeal spaces [167]. The DCs protective properties in AD seem to be predominantly linked to their ability to clear A*β* [168], as well as to cytokine and neurotrophin production and T cell activation [169, 170]. In the experiments of Ciramella and coworkers, in in vitro conditions, stimulation of monocyte-derived DCs (moDCs) with A*β*1–42 led to an increase in cell survival and soluble antigen uptake. Moreover, A*β* induced the elevated production of proinflammatory cytokines IL-1*β*, IL-6, and IL-18, and a decrease in MHC expression of the DCs, lowering the DCs ability to activate T cells [171]. Similar and even more pronounced results were obtained analyzing DCs collected from AD patients [169]. These results suggest that DCs contribute to brain damage probably by mechanisms of overactivation of inflammatory responses.

It is known that, in order to activate the adaptive immune response in brain, precursors of DCs migrate from the bone marrow to the CNS. There are two main subpopulations of circulating precursor DCs: one of them is myeloid cells (mDCs; lin−CD11c+ MHCIIhi, CD123lo) and the other is plasmacytoid cells (pDCs; lin−CD11c−MHCIImod, CD123hi) [172]. According to Bossù and collaborators, during AD progression, a significant decrease of myeloid DC precursors in the blood of AD patients was observed, which correlated with disease severity. These results suggest that the monitoring of blood DCs levels could be employed as a potential biomarker of AD progression. However, more studies are required to evaluate this possibility and to elucidate whether the reduction of blood DCs occurs in the results of their recruitment from the periphery to the brain or alterations of their differentiation from progenitor cells [173].

## 4. Interactions of T Cells and Amyloid *β*

It is known that, during AD progression, brain A*β* levels increase and A*β* is progressively deposited in the CNS where it can act as a specific antigen causing innate immune responses [174]. The A*β* can also act as an antigen activating adaptive responses, because mounting reports described A*β*-reactive circulating B cells in patients with AD [175]. The A*β*-reactive T cells in peripheral AD blood have also been described [112, 176]. Most recent studies by Monsonego and collaborators have shown that circulating A*β*-reactive T cells are present in patients with AD and that their levels in the blood increase with the disease's progression [112, 176]. Moreover, circulating A*β*-reactive T cells were detected at higher levels in AD patients after stimulation with the A*β*1–42 peptide compared to stimulation with A*β*1–40. Thus, these data indicate that the A*β*1–42 peptide is more immunogenic than A*β*1–40, in agreement with other studies showing a higher toxicity of A*β*1–42 than the A*β*1–40 peptide in the brain [177].

The localization of A*β* epitopes recognized by T cells is different than that of B cells. Epitopes recognized by CD4 T cells were identified in the C-terminal part of A*β*, that is, in the A*β*15–42 peptide, in contrast to dominant B cell epitopes identified in the A*β*1–15 peptide. More specifically, among the peptides that induced T cell proliferation, the A*β*16–30 peptide was the most effective. In addition, epitopes for T cells located in A*β*28–42 were specific to A*β*1–42 and not to A*β*1–40 which could explain the higher immunogenicity of A*β*1–42 than A*β*1–40.

A*β* is processed and presented by antigen-presenting cells (APCs) such as dendritic cells (DCs), in the context of MHC, and A*β*-specific T cell proliferation is mediated through MHC-TCR interactions [112, 176, 178, 179]. The question arises, where and how T cells are stimulated with the A*β* antigen. In light of data showing that in AD progression A*β* levels decrease in the CSF and there are no correlations of blood A*β* levels with AD severity, A*β* seems to be mainly captured by APCs located in the brain. Such MHCII high antigen-presenting cells either differentiate from brain-endogenous microglia or are recruited from the peripheral blood as a result of an increased expression of the CCL2 chemokine [157]. APCs located in the brain, such as perivascular and leptomeningeal dendritic cells, can present the antigen to T cells which infiltrate the AD brain [180–182]. T cell entry to the brain from the periphery is related mainly to A*β* deposition in the brain vasculature and to compromising the BBB stability and, as a result, a local inflammatory reaction [183, 184]. Low brain levels of IFN-*γ* were shown to promote T cell migration to the brain and also to regulate T cell adhesion, antigen presentation, expression, and signaling.

Another place of A*β* presentation by APCs to T cells is lymph nodes; brain APCs after capturing A*β* migrate next to lymph nodes, where they can induce T cell activation. Thus, it seems that the T cells reactivity towards A*β* could reflect to some extent an endogenous reaction to A*β* deposition in the brain in the context of the local innate immune response that occurs in AD [112, 183].

It was shown that A*β*-reactive T cells are maintained throughout life and increase with age in patients with AD [185]. Moreover, A*β*-reactive T cells can infiltrate the brain. However, the role of A*β* reactive T cells in AD is not obvious and in fact T cell reactivity to A*β* may cause either beneficial or injurious effects.

The beneficial effects of A*β* reactive T cells were indicated to be associated with A*β*1–42 as a self-antigen; self-reactive T cells were implicated in an immune regulation as well as brain repair processes during aging and in AD, but the particular mechanism is still debated [112, 176, 178, 179]. A*β*-reactive T cells seem to participate in numerous activities such as the release of regulatory cytokines [186] and an increase in the expression of neurotrophic factors in the brain [187, 188]. Recently it was shown that A*β*-reactive T cells are able to effectively target A*β* plaques in the brain and enhance the phagocytic activity of adjacent microglia via IFN-*γ*-induced TREM2 and SIRPb1 expression [176, 189–191]. In addition, IFN-*γ* facilitates T cell migration into and within the brain parenchyma [128] and promotes immune-regulatory processes [192, 193], as well as neuronal repair in the brain [168, 192]. Thus, anti-inflammatory cytokines such as IL-10 and TGF-*β*, together with a set of chemokines and neurotrophic factors secreted by T cells, prove therapeutic for the AD brain.

On the other hand, it was also shown that A*β*-reactive T cells can be boosted to promote pathogenic autoimmunity in AD. AD alterations in the T cells regulatory roles may lead to increased levels of proinflammatory cytokines such as IL-1*β*, TNF-*α*, and IL-6 and cause chronic inflammation which in turn enhances neurotoxicity and may impair key functions of the microglia in neuronal function and repair [194–196]. The conditions for neuroprotective versus promoting neurodegeneration role of T cells, and in particular A*β*-reactive T cells, in AD are still not fully elucidated and are possibly dependent on the AD stage, with more pronounced harmful function in more severe, later AD.

## 5. Cellular and Molecular Alterations in AD Peripheral Lymphocytes

Another line of research indicated molecular and cellular aberrations in blood lymphocytes from AD patients. The data shows changes in the distribution of different types of lymphocytes in the blood of AD patients [115] and the decline of immune functions due to decreased levels of T as well as B cells in peripheral blood [52]. Several reports confirmed the role of T cells in the development of AD-related abnormalities in the immune system, such as hyporesponsiveness of T cells to some intrinsic functional defects [50] and an increase of telomerase activity in lymphocytes that leads to diminishing lymphocyte proliferation activity and results in the loss of immune system functioning in AD patients [51–56]. Recently, we and other groups demonstrated alterations in the regulation of the cell cycle and oxidative stress response and impairment in mitochondrial functions in lymphocytes from AD blood (reviewed in [30]). However, in general little is known how molecular changes affect lymphocyte functions in the interplay between blood and brain in immune responses of AD patients and how they modify AD pathogenesis. Nevertheless, such changes were demonstrated early in AD in MCI patients and thus molecular alterations in blood lymphocytes seem promising as potential biomarkers for the early AD diagnostics using easily accessible tissue.

## 6. Conclusions and Future Perspectives

Summarizing, mounting evidence highlighted neuroinflammation as one of the key mechanisms in AD pathogenesis already at the early disease stage and in progression to later stages. Among factors which significantly contribute to AD inflammatory processes are immune mediators such as cytokines and chemokines, acting in the AD peripheral blood and brain, and T lymphocytes which migrate from peripheral blood through the BCSFB and the BBB to the AD brain. The inflammatory mediators present in the CSF and blood, as well as molecular and cellular alterations in peripheral lymphocytes in AD, represent potential biomarkers for diagnosing the early stages of AD and for monitoring progression to late stages. However, little is known on particular immune signatures characteristic for AD stages, mainly due to insufficiency in precise recruitment of patients for such studies. Further progress in biomarker development and immunotherapy requires determination of immune mediators characteristic for the early, moderate, and late AD stages.
